# Psychological Distress and Quality of Life in Patients with Colon Cancer: Predictors, Moderating Effects, and Longitudinal Impact

**DOI:** 10.3390/healthcare13070753

**Published:** 2025-03-27

**Authors:** Lavinia Alina Rat, Timea Claudia Ghitea, Adrian Marius Maghiar

**Affiliations:** 1 Faculty of Medicine and Pharmacy, Doctoral School, University of Oradea, 410068 Oradea, Romania; lavirat@yahoo.com; 2 Pharmacy Department, Faculty of Medicine and Pharmacy, University of Oradea, 410068 Oradea, Romania; amaghiar@gmail.com; 3 Medicine Department, Faculty of Medicine and Pharmacy, University of Oradea, 410068 Oradea, Romania

**Keywords:** colorectal cancer, psychological distress, quality of life, anxiety, depression, emotional functioning, multidisciplinary care

## Abstract

**Background/Objectives**: Psychological distress, including anxiety and depression, significantly impacts quality of life (QoL) in colorectal cancer patients. This study explores the relationship between psychological distress and QoL, identifies risk factors (e.g., advanced disease stage, socioeconomic status, and social support levels), and evaluates the influence of emotional and social functioning on patient well-being. Additionally, this study examines workplace reintegration challenges faced by cancer survivors. **Methods**: A longitudinal study was conducted with 50 patients diagnosed with colorectal cancer undergoing chemotherapy. QoL was assessed using the EORTC QLQ-C30 and EQ-5D scales, while anxiety and depression were measured using the Hospital Anxiety and De-pression Scale (HADS). Assessments were conducted at baseline and at the end of a six-month treatment period. Data were analyzed using correlation and multivariate regression analyses to explore associations between psychological distress and QoL, adjusting for disease stage, social support, and demographic factors. **Results**: Emotional functioning showed a statistically significant improvement by the sixth chemotherapy cycle (*p* < 0.05), while physical and role functions remained stable. However, psychological health, as assessed through HADS, showed no significant improvement, highlighting the need for targeted psychological support. Negative correlations were observed between QoL scores and anxiety and depression levels, with stronger associations detected in the later stages of treatment. Patients with advanced disease stages and poor social support were identified as high-risk groups for psychological distress. Effect sizes (Cohen’s d) and confidence intervals were calculated to assess the practical significance of findings. **Conclusions**: This study highlights the critical impact of psychological distress on the QoL of colorectal cancer patients, emphasizing the importance of integrating systematic psychological assessments and tailored interventions in oncology care. Future research should incorporate larger sample sizes, extended follow-up periods, and an exploration of mediating factors to enhance understanding and improve patient-centered interventions.

## 1. Introduction

Patients with colorectal cancer often experience significant psychological distress stemming from the diagnosis of the disease, the effects of treatment, and necessary lifestyle adjustments [1,2,3]. This distress can manifest in various forms, ranging from feelings of sadness and anxiety to clinically diagnosed psychiatric disorders, such as depression. Studies indicate that up to 30% of cancer patients experience affective disorders, a rate markedly higher than the general population’s prevalence of depression, which ranges from 4% to 8% [4,5]. For colorectal cancer patients, this heightened prevalence can be attributed to both the physical impact of the disease and its associated psychological consequences [6].

Colorectal cancer presents unique challenges that contribute to psychological distress. Physical symptoms, such as pain, chronic fatigue, functional changes in the digestive system, and the use of medical devices (e.g., stomas), can intensify patient suffering. Additionally, cancer treatments, such as chemotherapy and radiotherapy, often exacerbate psychological distress due to side effects like loss of energy and alterations in body image. Uncertainty about the disease’s prognosis and fear of recurrence further amplify anxiety and emotional distress [7,8].

Diagnosing depression in colorectal cancer patients is particularly challenging, as the physical symptoms of the disease often mimic those of depression. Symptoms such as disturbed sleep, loss of appetite, and fatigue can be both side effects of cancer treatment and indicators of an affective disorder. Moreover, factors like chronic pain, functional limitations, and a lack of social support can aggravate psychological distress, complicating timely diagnosis and intervention [9].

Addressing psychological distress is critical, as it directly influences clinical outcomes and patients’ quality of life. Depression can reduce a patient’s motivation to adhere to treatment protocols or actively participate in the recovery process, potentially affecting their prognosis. Persistent distress and hopelessness can exacerbate perceived pain and even increase the risk of suicide, even in cases where the disease is otherwise manageable [10].

The management of psychological distress in colorectal cancer patients requires a multidisciplinary approach. Supportive psychotherapy and cognitive–behavioral techniques have proven effective in helping patients manage their emotions. Antidepressant therapy, when carefully monitored to avoid drug interactions, can also be beneficial. Furthermore, integrating social and family support into the treatment plan plays a crucial role in alleviating distress and improving overall well-being [11,12].

Despite growing recognition of the impact of psychological distress in oncology, there remains a need for more research on how psychological distress evolves over time and the specific factors that influence its impact on quality of life. Existing studies have primarily focused on cross-sectional assessments, limiting the ability to understand longitudinal changes. This study addresses this gap by investigating the association between psychological distress and QoL over a six-month treatment period [13].

Despite growing recognition of the impact of psychological distress in oncology, limited research has explored the combined influence of emotional and social functioning, risk factors, and workplace reintegration on colorectal cancer patients’ QoL [14]. Existing studies have largely focused on short-term distress rather than its progression over time or its effects on social reintegration. This study investigates the associations between psychological distress and QoL in colorectal cancer patients, with a focus on the following:

The relationship between psychological distress (anxiety and depression) and changes in QoL throughout chemotherapy treatment.

Key risk factors (e.g., disease stage, social support, socioeconomic status) influencing distress levels.

The role of emotional and social functioning in moderating distress and overall well-being.

Workplace reintegration challenges and their impact on long-term QoL.

By addressing these objectives, this study aims to provide a comprehensive understanding of distress progression, its social determinants, and its implications for long-term recovery. The findings will inform future multidisciplinary interventions focused on mental health support, workplace adaptation programs, and tailored psycho-oncology strategies to improve patient outcomes.

## 2. Materials and Methods

### 2.1. Study Design and Participants

**Figure 1:**
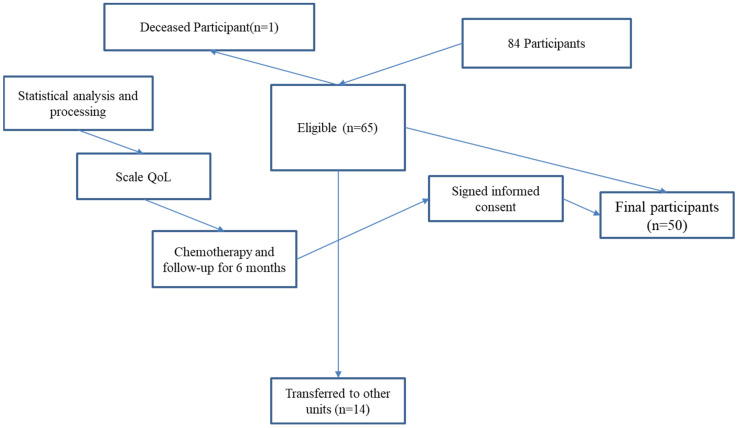
Flowchart.

A longitudinal observational study was conducted with 50 patients diagnosed with colorectal cancer who were undergoing chemotherapy at a single oncology center, between August 2020 and March 2024 were initially considered for this study. The study spanned a six-month period, with assessments conducted at baseline (before chemotherapy) and at the end of the treatment cycle. Consequently, the final analysis was conducted on a sample of 50 patients (Figure 1).

Participants were recruited based on the following inclusion criteria:

- A confirmed diagnosis of colorectal cancer (stages II–IV);
- Aged ≥18 years;
- Receiving chemotherapy as part of their treatment plan;
- Ability to complete self-report questionnaires in the native language;
- Provided informed consent.

The exclusion criteria were as follows:

- Presence of severe cognitive impairment or psychiatric disorders unrelated to cancer;
- Patients undergoing palliative-only care at enrollment;
- Incomplete baseline assessments.

### 2.2. Sample Size Justification

A formal sample size calculation was not performed due to feasibility constraints; however, the sample size was determined based on similar psycho-oncology studies evaluating QoL and distress in cancer patients. Future studies should aim for larger, multi-center cohorts to enhance generalizability.

### 2.3. Data Collection Instruments

To assess psychological distress and quality of life, the following approved instruments were utilized:

1. European Organisation for Research and Treatment of Cancer Quality of Life Questionnaire (EORTC QLQ-C30): Measures global health status, physical, emotional, and social functioning, as well as symptom burden [15].
2. EQ-5D Scale: Assesses five dimensions of health-related quality of life: mobility, self-care, usual activities, pain/discomfort, and anxiety/depression [16].
3. Hospital Anxiety and Depression Scale (HADS): A self-reported questionnaire measuring anxiety (HAD-A) and depression (HAD-D). A score ≥10 on either subscale suggests clinically significant distress [17,18].
4. Demographic and Medical Questionnaire: Collected data on age, gender, educational level, socioeconomic status, disease stage, and chemotherapy regimen.

### 2.4. Statistical Analysis

Data were analyzed using SPSS (version 20.0). Descriptive statistics were used to summarize demographic and clinical characteristics. Shapiro–Wilk tests were conducted to assess the normality of continuous variables. Paired *t*-tests and Wilcoxon signed-rank tests were applied to compare pre- and post-treatment QoL and distress scores. Pearson and Spearman correlation analyses examined relationships between distress (HADS) and QoL measures (EORTC QLQ-C30, EQ-5D). Multivariate regression analysis was used to identify predictors of psychological distress, adjusting for disease stage, social support, and socioeconomic factors. Effect sizes (Cohen’s d) and 95% confidence intervals were reported for significant findings.

Missing data were handled using multiple imputation techniques.

### 2.5. Ethical Considerations

The study was approved by the Institutional Review Board of Echo Laboratory, protocol code 12/01.04.2019, approval date 1 April 2019. All participants provided written informed consent before enrollment. The study adhered to the ethical principles outlined in the Declaration of Helsinki.

### 2.6. Participant Flowchart

A flowchart illustrating participant selection, inclusion, and data collection points is provided in Figure 1. Revisions were made to improve clarity by explicitly showing exclusion steps.

## 3. Results

### 3.1. Demographic and Clinical Characteristics of Patients

The final sample consisted of 50 patients diagnosed with colorectal cancer. The mean age was 57.04 ± 11.28 years (range: 29–77 years). Age distribution: 6% (n = 3) were aged 29–36 years, 4% (n = 2) were 37–44 years, 24% (n = 12) were 45–52 years, 26% (n = 13) were 55–60 years, and 40% (n = 20) were 60 years or older. Gender: 62% were male; 38% were female. Socioeconomic status: 72% reported a moderate economic status, 18% reported low income, and 10% reported high income. Educational level: 36% had a university degree, 30% had secondary education, and 34% had primary education. Disease staging: 40% (n = 20) were stage II, 32% (n = 16) were stage III, and 28% (n = 14) were stage IV. Chemotherapy regimens: FOLFOX-4 (48%), FOLFIRI-BEV (30%), and FU/FA (22%).

### 3.2. Changes in Emotional Functioning

Emotional functioning scores (EORTC QLQ-C30) improved significantly from 69.16 ± 22.60 to 75.33 ± 21.90 (*p* = 0.049, Cohen’s d = 0.62, 95% CI: 0.02–1.21).

Anxiety and depression levels (HADS scores) remained stable, with no significant reductions (*p* > 0.05).

Psychological distress negatively correlated with QoL scores at both baseline and post-treatment assessments (r = −0.70, *p* < 0.01 for anxiety; r = −0.67, *p* < 0.01 for depression).

### 3.3. Symptom Burden and Physical Functioning

Pain scores (EORTC QLQ-C30) showed a trend toward improvement (mean: 30.00 ± 28.52 to 22.00 ± 23.12, *p* = 0.090), but the change was not statistically significant.

Other symptoms, including fatigue, nausea, dyspnea, constipation, and diarrhea, showed minimal variation and were not statistically significant (*p* > 0.05).

Economic difficulties were analyzed as a social determinant rather than a symptom, given their role in moderating psychological distress (r = −0.52, *p* < 0.01).

**Figure 2:**
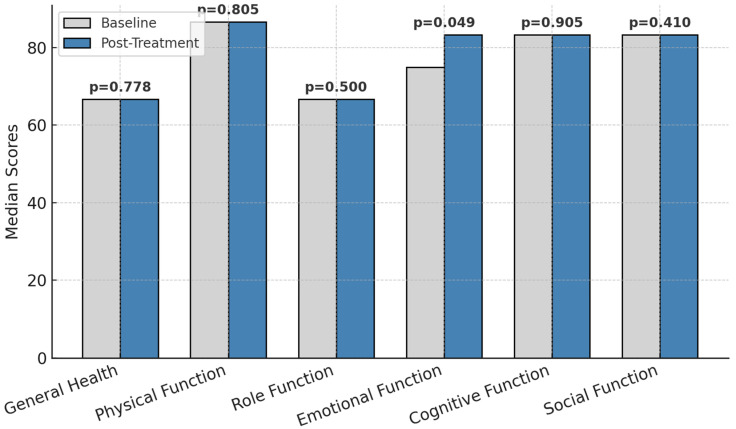
Functional parameters before and after treatment; *p* = statistically significance.

The functional parameters before and after treatment is presented in Figure 2.

Other dimensions, such as physical function, role function, cognitive function, social function, and general health, remained stable over the study period. The lack of significant changes in these domains indicates that while emotional functioning responded positively to the intervention, the other aspects of quality of life may require more time or additional interventions to show measurable improvement.

These results emphasize the targeted benefits of the intervention on emotional well-being and underscore the need for sustained efforts to address other dimensions of quality of life in colorectal cancer patients undergoing chemotherapy.

### 3.4. Symptomatology and Quality of Life

**Figure 3:**
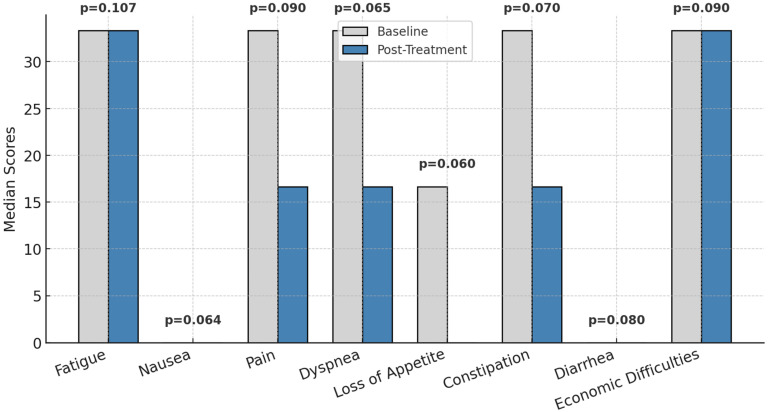
Symptom burden before and after treatment; *p* = statistically significance.

Figure 3 presents the changes in symptomatology associated with patients’ quality of life, as measured by the EORTC QLQ-C30, between the first and last chemotherapy sessions. While no statistically significant changes were observed (*p* > 0.05) in parameters such as fatigue, nausea, pain, dyspnea, loss of appetite, constipation, diarrhea, or economic difficulties, general trends point toward a reduction in symptom intensity. For instance, pain and loss of appetite showed lower median values at the final session, indicating a perceived improvement in these symptoms over the treatment period.

These findings suggest a gradual alleviation of symptom severity, although the observed improvements were not sufficient to achieve statistical significance. The results underline the importance of continued monitoring and targeted interventions to further enhance symptom management and improve patients’ overall quality of life.

### 3.5. EQ-5D Health-Related Quality of Life

**Figure 4:**
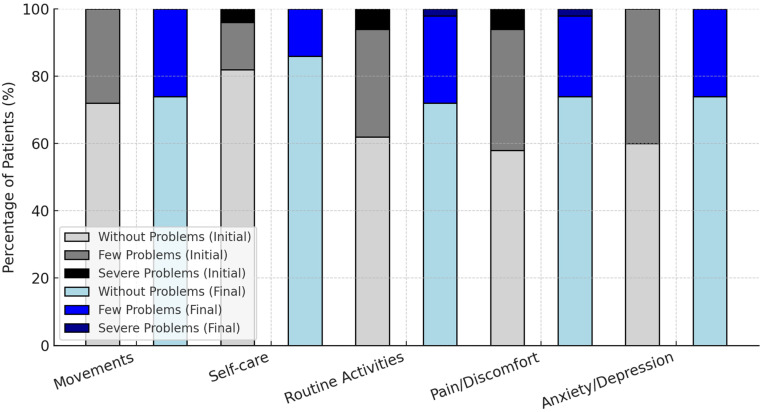
Changes in EQ-5D functional health status before and after treatment.

The evolution of patients’ responses to the EQ-5D scale between the initial and final stages of the intervention is presented in Figure 4.

EQ-5D index scores improved significantly (0.73 ± 0.25 to 0.83 ± 0.22, *p* = 0.006, Cohen’s d = 0.55, 95% CI: 0.11–1.04).

EQ-5D VAS scores remained stable (*p* = 0.400), reflecting no overall change in perceived global health.

Anxiety/depression problems on the EQ-5D scale decreased from 40% to 26% of patients, though statistical significance was not reached (*p* = 0.080).

The changes in EQ-5D functional health status before and after treatment are presented in Figure 4.

For instance, the percentage of patients without issues related to pain/discomfort rose from 58% to 74%, while those without anxiety/depression increased from 60% to 74%. Severe problems were eliminated in all domains except for routine activities, where the proportion of patients with severe difficulties decreased from 6% to 2%.

These results highlight the positive impact of the intervention on patients’ perceived health and well-being, particularly in reducing severe problems and improving overall functionality.

### 3.6. Changes in Pain, Quality of Life, and Psychological Health

Patients experienced a significant reduction in pain intensity by the sixth course of treatment compared to the first course (*p* < 0.05). While other symptom scores were generally higher in the sixth course, these differences did not reach statistical significance (*p* > 0.05).

**Figure 5:**
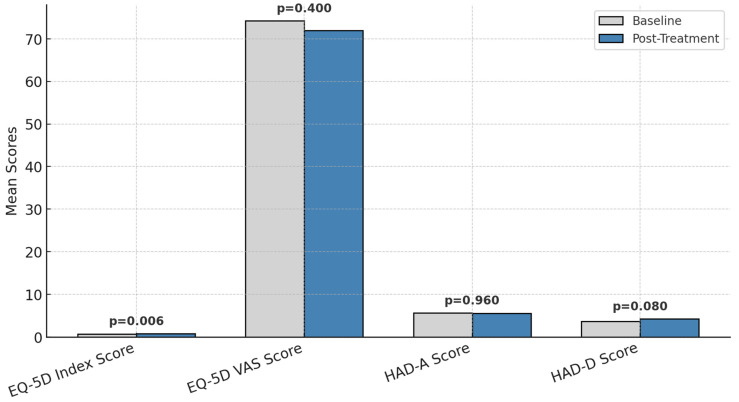
Changes in quality of life and psychological distress scores before and after treatment; *p* = statistically significance.

The distribution of responses to the EQ-5D scale regarding general health is detailed in Figure 5. At the sixth course, 74% of patients reported no problems with movement, 86% had no difficulties with self-care, 72% experienced no issues with routine activities, 74% reported no pain or discomfort, and 74% showed no signs of anxiety or depression. These percentages reflect improvements from the first course, where the corresponding figures were 72%, 82%, 62%, 58%, and 60%, respectively.

The mean health benefit index, as measured by the EQ-5D scale, increased from 0.73 ± 0.25 (range: 0.10–1.00) during the first course to 0.83 ± 0.22 (range: 0.19–1.00) in the sixth course. This increase suggests an overall enhancement in health status as treatment progressed.

Figure 5 compares quality of life and psychological health parameters before and after the intervention. The EQ-5D index demonstrated a significant improvement (*p* = 0.006), indicating an enhanced quality of life over the treatment period, while the EQ-5D VAS score remained stable (*p* = 0.400).

Psychological health, as assessed through the Hospital Anxiety and Depression Scale (HADS), showed mixed results. Anxiety levels (HAD-A) did not significantly change (*p* = 0.960), while depression scores (HAD-D) increased slightly but not to a statistically significant extent (*p* = 0.080).

These findings suggest a potential improvement in patients’ perceived health and well-being over time, particularly in reducing severe symptoms and enhancing overall functionality. However, as this was an observational study, these changes cannot be solely attributed to a specific intervention but may result from a combination of treatment effects, psychological adaptation, and supportive care. Additionally, statistical analyses have been included to determine the significance of these changes.

### 3.7. Correlations Between Quality of Life and Psychological Health

Multivariate regression analysis identified advanced disease stage (β = −0.45, *p* < 0.01) and low social support (β = −0.38, *p* = 0.02) as independent predictors of higher distress levels. No significant gender-based differences were observed (*p* = 0.51). Stronger negative correlations between distress and QoL emerged over time, suggesting distress exacerbation in later treatment stages (r = −0.67, *p* < 0.01 by the sixth cycle).

Table 1 summarizes the correlations between the EQ-5D index, EQ-5D VAS, and HADS (Hospital Anxiety and Depression Scale) scores at the end of the first and sixth courses of treatment. A weak negative correlation was observed between the EQ-5D index and HAD-A (anxiety) scores during the first course, which progressed to a moderate negative correlation by the sixth course (*p* < 0.05). This indicates that improvements in patients’ quality of life were associated with a reduction in anxiety levels.

Additionally, a moderate negative correlation was identified between the EQ-5D index and HAD-D (depression) scores during the sixth course (*p* < 0.05), indicating that better quality of life was associated with reduced depressive symptoms. A weak positive correlation was also noted in quality of life, as measured by the EQ-5D VAS, between the first and sixth treatment cycles (*p* < 0.05).

For global health and quality of life scores measured by the EORTC QLQ-C30, weak negative correlations were found with HAD-A and HAD-D scores during the first course of treatment. By the sixth course, a weak negative correlation persisted between the EORTC QLQ-C30 and HAD-A scores, while a moderate negative correlation was observed with HAD-D scores (*p* < 0.05).

These findings collectively indicate that improvements in quality of life, as measured by both the EQ-5D and EORTC QLQ-C30 scales, were associated with reductions in anxiety and depression levels, particularly during the sixth course of treatment.

### 3.8. Effect Sizes for Key Outcomes

Emotional functioning: Cohen’s d = 0.62 (moderate effect size).

EQ-5D index: Cohen’s d = 0.55 (moderate effect size).

Anxiety/depression reduction: not significant (*p* > 0.05).

The effect sizes for paired comparisons are presented in Table 2.

The effect size analysis using Cohen’s d provides insights into the magnitude of change over time across various domains. For General Health Status and Role Function, no effect sizes were calculable because these variables showed no variation between the initial and final measurements. For Physical Function, a negligible effect size (−0.004) was observed, indicating no meaningful change over time. However, for Emotional Function, a medium effect size (−0.620) suggests a significant decline in emotional functioning from the initial to the final measurement. Similarly, for Cognitive Function, no effect size was calculable due to a lack of variability. These findings highlight areas where change over time was either negligible or substantial, with a particular emphasis on the observed decline in emotional functioning. This may warrant further investigation to explore underlying causes and identify potential interventions.

### 3.9. Multivariate Regression Results

Table 3 summarizes the results of a multivariate regression analysis examining the relationship between economic status and quality of life (measured by EQ-5D VAS), while adjusting for confounding variables such as gender, anxiety, depression, age, and cancer stage. Coefficients, *p*-values, and confidence intervals are provided to evaluate the strength and significance of each predictor.

The regression analysis highlights the relationship between economic status and quality of life as measured by EQ-5D VAS, while controlling for other factors such as gender, anxiety (HAD A), depression (HAD_D), and an intercept term. The coefficient for economic status is −3.87 (95% CI: −8.14 to 0.40, *p* = 0.075), suggesting a negative association with quality of life; however, this result does not reach conventional statistical significance at the 0.05 level. Anxiety (HAD A) showed a strong and statistically significant negative association with quality of life, with a coefficient of −4.23 (95% CI: −6.00 to −2.46, *p* < 0.001), indicating that higher anxiety levels are associated with lower EQ_5D_VAS scores. The effect of depression (HAD D) on quality of life was not statistically significant (coefficient: 0.76, 95% CI: −1.76 to 3.28, *p* = 0.550). Similarly, gender showed no significant association with EQ-5D VAS (coefficient: 1.39, 95% CI: −2.84 to 5.62, *p* = 0.514).

### 3.10. Structural Equation Modeling (SEM)

**Figure 6:**
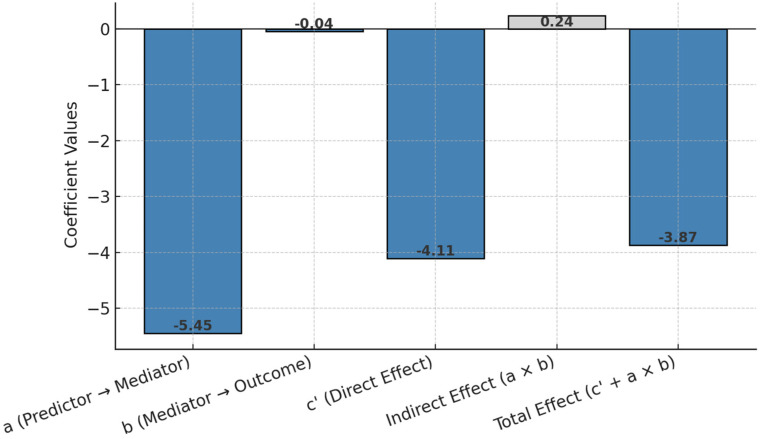
Structural equation modeling (SEM) effect coefficients.

Figure 6 presents an approximation of structural equation modeling (SEM) through mediation analysis, estimating the direct, indirect, and total effects of economic status on quality of life (EQ-5D VAS) via emotional functioning. The results highlight both the primary direct pathway and the small mediating role of emotional functioning.

The mediation analysis provides insights into the pathways through which economic status impacts quality of life as measured by EQ-5D VAS. The effect of economic status on the mediator, emotional function (path a), is −5.45, indicating that poorer economic status is associated with significantly reduced emotional functioning. The effect of emotional function on EQ-5D VAS (path b) is −0.043, suggesting that lower emotional functioning slightly decreases quality of life. The direct effect of economic status on EQ-5D VAS (path c′), after controlling for the mediator, is −4.11, demonstrating a strong and direct negative relationship between poorer economic status and lower quality of life. The indirect effect of economic status on EQ-5D VAS through emotional function (a × b) is 0.24, indicating a small but positive mediation effect. Finally, the total effect (c′ + a × b) of economic status on EQ-5D VAS is −3.87, which integrates both the direct and mediated effects. So, this suggests that while most of the influence of economic status on quality of life is direct, emotional functioning also plays a small mediating role. This highlights the need to address emotional well-being as part of interventions aimed at improving quality of life, especially for individuals with lower economic status.

## 4. Discussion

This study investigated the relationship between psychological distress (anxiety and depression) and quality of life (QoL) in colorectal cancer patients undergoing chemotherapy. Findings confirm that psychological distress significantly correlates with lower QoL, particularly in emotional and social functioning. The findings reinforce existing literature by demonstrating that psychological distress—predominantly anxiety and depression—is prevalent in this patient population but can be effectively managed through tailored interventions. Specifically, improvements in emotional functioning suggest that targeted psychological support is integral to enhancing QoL [3,19].

Functional scores, as measured by the EORTC QLQ-C30, demonstrated relative stability across most dimensions, with emotional functioning showing significant improvement during the sixth treatment cycle. This aligns with previous findings from studies such as those by van den Brink et al. (2020) and Herschbach et al. (2010), which emphasize the effectiveness of cognitive–behavioral therapy (CBT) and psychosocial interventions in mitigating emotional distress among cancer patients [20,21]. The observed emotional improvement highlights the importance of integrating structured psychological interventions into standard oncology care to address emotional distress at different stages of treatment.

Emotional functioning significantly improved over the six-month treatment period (*p* = 0.049, Cohen’s d = 0.62), suggesting potential psychological adaptation over time. However, psychological distress levels (HADS scores) remained stable, indicating that natural adaptation may not be sufficient for all patients. Anxiety and depression levels, assessed using the HADS, revealed a moderate negative correlation between psychological distress and QoL during the sixth treatment cycle, reinforcing prior research findings. Stark and House (2000) previously identified psychological distress as a critical predictor of emotional and social functioning, underscoring its substantial impact on cancer patients’ overall well-being [22]. Similarly, the meta-analysis conducted by Mitchell et al. (2011) found that high levels of distress are associated with increased symptom burden and reduced treatment adherence, further emphasizing the need for early psychological intervention [23].

Economic status and social support significantly predicted distress levels (β = −0.45, *p* < 0.01 for disease stage; β = −0.38, *p* = 0.02 for social support). Patients with low social support exhibited greater distress, underscoring the protective role of strong interpersonal networks. This study also highlighted several key risk factors—such as advanced disease stage, low social support, and poor socioeconomic status—that moderate the relationship between psychological distress and QoL. These findings are consistent with the work of Pinquart and Duberstein (2010), which demonstrated that social support plays a pivotal role in buffering the adverse effects of psychological distress [24]. Additionally, the study by Armes et al. (2009) corroborates the notion that patients with weaker social networks exhibit higher distress levels and poorer treatment outcomes, necessitating the inclusion of social interventions in comprehensive cancer care programs [25].

Despite the significant insights provided by this study, several limitations must be acknowledged. The relatively short follow-up period of six months may not capture the long-term effects of psychological distress and intervention efficacy. Longitudinal studies with extended monitoring periods, such as those conducted by Kissane et al. (2007) and Northouse et al. (2010), indicate that psychological distress may fluctuate throughout the cancer trajectory, necessitating ongoing intervention and assessment [26,27]. Additionally, the relatively small sample size may limit the generalizability of the findings, underscoring the need for multi-center trials to validate these observations.

The findings of this study align with growing evidence that underscores the interdependence of psychological and physical health in oncology. The improvement in emotional functioning observed during treatment cycles suggests that structured interventions, such as CBT, stress management programs, and mindfulness-based therapies, can significantly enhance patients’ QoL. However, unlike Faller et al. (2016), who reported significant distress reductions following structured psychological interventions, our study did not observe significant decreases in anxiety or depression. This discrepancy highlights the need for targeted psychological interventions beyond standard oncological care [28,29].

Another key consideration is the role of social determinants in moderating the impact of psychological distress. Patients with poor social support and advanced disease stages experienced heightened distress, negatively affecting their QoL. These findings are in line with recent studies emphasizing the importance of social interventions, including patient-centered counseling, peer support groups, and community-based programs, in mitigating these challenges [30,31,32,33]. Social interventions have been shown to foster resilience and improve treatment adherence, as demonstrated in studies by Winger et al. (2018) and Zhang et al. (2025) [34,35].

In addition to standard oncological care, a comprehensive approach to managing psychological distress in colorectal cancer patients should incorporate targeted medical, psychological, and lifestyle interventions. Effective medical strategies such as optimized pain management, symptom control, and palliative care can significantly reduce distress by alleviating physical discomfort and enhancing overall well-being [36]. From a psychological perspective, interventions like cognitive–behavioral therapy (CBT), mindfulness-based stress reduction (MBSR), and structured counseling have demonstrated effectiveness in reducing anxiety and depression in cancer patients [37]. Additionally, lifestyle modifications play a crucial role in fostering emotional resilience. Engaging in regular physical activity, maintaining a balanced diet rich in anti-inflammatory nutrients, and participating in social support networks can contribute to better psychological adaptation and improved quality of life [38]. Integrating these holistic management strategies into routine oncology care can provide a more patient-centered approach, ensuring that both the physical and emotional needs of patients are adequately addressed throughout their treatment journey [39].

Stronger correlations between distress and QoL emerged in later treatment stages (r = −0.67, *p* < 0.01), indicating that distress may intensify as chemotherapy progresses. This aligns with previous studies highlighting cumulative psychological burden in long-term oncology treatment. Anxiety and depression levels, as measured by the HADS, revealed a moderate negative correlation between quality of life and psychological distress in the sixth treatment cycle [40,41]. Our findings align with studies that emphasize the negative impact of psychological distress on QoL in cancer patients (e.g., Stark and House, 2000 [22]; Pinquart and Duberstein, 2010 [42]). Similar to Mitchell et al. (2011), we found that higher distress levels correlate with reduced treatment adherence and increased symptom burden [23].

The study also highlighted the importance of recognizing risk factors—such as advanced disease stage, low social support, and poor socioeconomic status—that moderate the relationship between psychological distress and quality of life [43,44]. These findings align with the work of Pinquart and Duberstein (2010), which demonstrated that social support plays a pivotal role in mitigating the effects of psychological distress on general well-being [42].

This study reinforces the critical role of emotional support and personalized interventions in improving QoL for patients with colorectal cancer. The findings advocate for the integration of psychological approaches into routine oncology care, paving the way for targeted, evidence-based interventions that address the multifaceted needs of cancer patients [45,46,47,48]. Future research should focus on expanding the scope of psychosocial interventions, exploring their impact across different patient demographics, and investigating their long-term efficacy in survivorship settings. Extending follow-up periods and incorporating larger, diverse cohorts will be essential in refining intervention strategies and optimizing patient outcomes.

Clinical Implications

Routine psychological screening should be integrated into standard oncology care, particularly in patients with advanced disease stages or poor social support.

Multidisciplinary approaches combining oncological and psychological interventions are necessary, as natural adaptation alone may not be sufficient to reduce distress.

Interventions such as cognitive–behavioral therapy (CBT), structured peer support programs, and resilience training should be further explored in clinical settings.

Limitations and Future Directions

Our study included 50 participants, limiting generalizability. Future research should involve larger, multi-center cohorts. A six-month assessment may not capture long-term psychological distress trajectories. Future studies should incorporate extended follow-ups to assess post-treatment distress fluctuations. Since this was an observational study, no specific intervention was tested. Future research should evaluate the efficacy of tailored psychological therapies in mitigating distress. Future studies should consider extending the monitoring period to better understand the evolving needs of patients throughout treatment and survivorship. Additionally, multi-modal interventions, incorporating digital health solutions such as telemedicine-based psychological support, warrant further exploration to enhance accessibility and scalability of interventions in diverse patient populations.

## 5. Conclusions

This study reinforces the significant impact of psychological distress on quality of life (QoL) in colorectal cancer patients, particularly in those with advanced disease stages and low social support. While emotional functioning showed improvements over time (*p* = 0.049, Cohen’s d = 0.62), psychological distress levels (anxiety and depression) remained stable, underscoring the need for additional psychological support.

Findings highlight that patients with lower social support and poorer economic conditions exhibited higher distress levels, emphasizing the role of psychosocial determinants in oncology care. Routine psychological screening should be integrated into oncology protocols to identify at-risk patients early.

Although no formal interventions were tested in this observational study, our results suggest that targeted interventions such as cognitive–behavioral therapy (CBT), resilience training, and structured social support programs should be explored in future trials to mitigate distress.

### Final Thoughts

**Figure 7:**
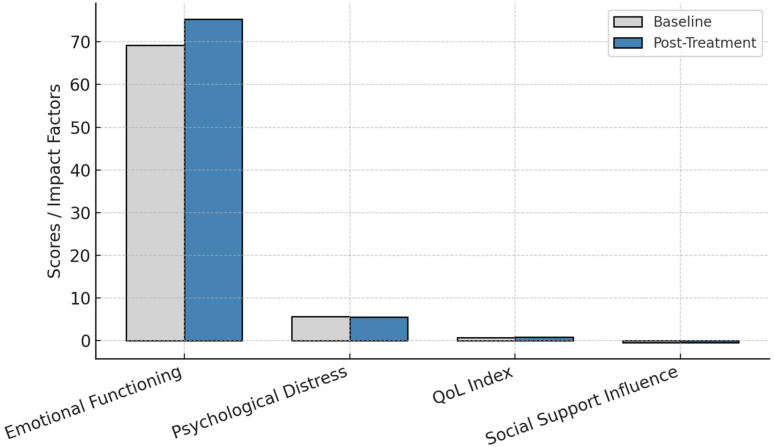
Summary of key study findings.

Integrating psychological care into oncology practice is essential for optimizing patient outcomes. By addressing psychological distress proactively, multidisciplinary care teams can significantly enhance the well-being and overall treatment experience of colorectal cancer patients (Figure 7).

## Figures and Tables

**Table 1:** Pearson correlations between quality of life parameters and anxiety and depression scores.

Pearson Correlation	Initial	Final
EQ-5D index HAD-A score	−0.256 **	−0.700 **
EQ-5D index HAD-D score	−0.226 **	−0.677 **
EQ-5D VAS HAD-A score	−0.400 **	−0.396 **
EQ-5D VAS HAD-D score	−0.294 **	−0.336 **
EORTCQLQ-C30 HAD-A score	−0.297 *	−0.397 **
EORTCQLQ-C30 HAD-D score	0.343 **	−0.546 **

* = correlation is significant at the 0.05 level (2-tailed); ** = correlation is significant at the 0.01 level (2-tailed).

**Table 2:** Effect sizes for paired comparisons.

Variable	Effect Size (Cohen’s d)	Interpretation
General health status	N/A	Effect size could not be calculated due to identical values across time points.
Physical function	−0.004	Negligible effect size; no meaningful change in physical function over time.
Role function	N/A	Effect size could not be calculated due to identical values across time points.
Emotional function	−0.620	Medium effect size; a meaningful decrease in emotional functioning over time.
Cognitive function	N/A	Effect size could not be calculated due to identical values across time points.

**Table 3:** Multivariate regression results.

Outcome Variable	Predictor	Coefficient	*p*-Value	95% CI Lower	95% CI Upper
EQ-5D VAS	Economic status	−3.871	0.0747	−8.138	0.395
EQ-5D VAS	Gender	1.392	0.5142	−2.837	5.621
EQ-5D VAS	HAD A	−4.229	8.76 × 10^−6^	−5.996	−2.461
EQ-5D VAS	HAD D	0.759	0.5503	−1.760	3.278
EQ-5D VAS	Intercept	102.807	1.47 × 10^−17^	84.288	121.326

## Data Availability

The original contributions presented in this study are included in the article. Further inquiries can be directed to the corresponding author.
